# A deep generative adversarial network capturing complex spiral waves in disinhibited circuits of the cerebral cortex

**DOI:** 10.1186/s12868-023-00792-6

**Published:** 2023-03-24

**Authors:** Megan Boucher-Routhier, Jean-Philippe Thivierge

**Affiliations:** 1 School of Psychology, University of Ottawa, 156 Jean-Jacques Lussier, Ottawa, ON K1N 6N5 Canada; 2 University of Ottawa Brain and Mind Research Institute, 451 Smyth Rd., Ottawa, ON K1H 8M5 Canada

**Keywords:** Deep neural network, Multi electrodes, Epilepsy, Complexity

## Abstract

**Background:**

In the cerebral cortex, disinhibited activity is characterized by propagating waves that spread across neural tissue. In this pathological state, a widely reported form of activity are spiral waves that travel in a circular pattern around a fixed spatial locus termed the center of mass. Spiral waves exhibit stereotypical activity and involve broad patterns of co-fluctuations, suggesting that they may be of lower complexity than healthy activity.

**Results:**

To evaluate this hypothesis, we performed dense multi-electrode recordings of cortical networks where disinhibition was induced by perfusing a pro-epileptiform solution containing 4-Aminopyridine as well as increased potassium and decreased magnesium. Spiral waves were identified based on a spatially delimited center of mass and a broad distribution of instantaneous phases across electrodes. Individual waves were decomposed into “snapshots” that captured instantaneous neural activation across the entire network. The complexity of these snapshots was examined using a measure termed the participation ratio. Contrary to our expectations, an eigenspectrum analysis of these snapshots revealed a broad distribution of eigenvalues and an increase in complexity compared to baseline networks. A deep generative adversarial network was trained to generate novel exemplars of snapshots that closely captured cortical spiral waves. These synthetic waves replicated key features of experimental data including a tight center of mass, a broad eigenvalue distribution, spatially-dependent correlations, and a high complexity. By adjusting the input to the model, new samples were generated that deviated in systematic ways from the experimental data, thus allowing the exploration of a broad range of states from healthy to pathologically disinhibited neural networks.

**Conclusions:**

Together, results show that the complexity of population activity serves as a marker along a continuum from healthy to disinhibited brain states. The proposed generative adversarial network opens avenues for replicating the dynamics of cortical seizures and accelerating the design of optimal neurostimulation aimed at suppressing pathological brain activity.

**Supplementary Information:**

The online version contains supplementary material available at 10.1186/s12868-023-00792-6.

## Background

In disinhibited cortical circuits, neural activity is characterized by patterns that propagate across widespread networks [1]. These patterns take on different forms, including planar waves traveling in a single direction, saddle waves emerging from the interaction between multiple sites of propagation, and spiral waves that evolve in a circular motion around a fixed spatial locus [2–8]. These spiral waves are found during interictal epileptic activity [9–12] and are reported in cortical networks both in vitro [1] and in vivo [13]. Their origin and characteristics, however, remain to be fully elucidated, as they constitute rare events relative to background activity and cannot be captured by simple computational models including classic balanced excitation/inhibition networks [14].

A promising avenue to describe patterns of activity is to examine their *complexity*, indicative of the number of distinct factors needed to capture neural fluctuations. In many instances, the activity of large networks can be closely approximated using only a small number of factors that capture much of the variance across neurons [2]. This low complexity suggests that a few broad features, such as oscillations or shared patterns of fluctuation, may explain most population-level activity, thus greatly simplifying descriptions of neural dynamics and providing a strong guidance to theories of brain function [15–17].

While alterations in neural complexity are expected in disinhibited brain networks [18, 19], diverging lines of evidence point to either an increase or decrease in complexity, thus leaving unresolved the relation between complexity and pathological brain states. Previous work suggests that pathologically disinhibited states are accompanied by a decrease in complexity given that they exhibit highly stereotypical forms of activity. More specifically, disinhibiting cortical neurons by blocking GABA_A_ transmission increases synchronization and reduces the complexity of oscillations [18, 19]. Other work, however, suggests that disinhibited waves contribute to an increase in neural complexity as they form intricate patterns that extend both in time and across neuronal tissue [14, 20, 21]. Examining the complexity of spiral waves is key to disambiguating these viewpoints.

In this work, we studied cortical population activity in disinhibited slices recorded with a high-density multi-electrode array (HD-MEA) [22]. Disinhibited neural activity exhibited spiral waves whose amplitude was concentrated in the delta frequency range (1–4 Hz). These waves were analyzed by extracting “snapshots” that captured the instantaneous neural activation across whole cortical networks. The complexity of these snapshots was analyzed using a measure termed the participation ratio (PR) [23–26].

To capture spiral waves and account for their complexity, a deep generative adversarial network (GAN) was trained to generate snapshots of activity that matched those obtained experimentally [27]. After training, the GAN model produced synthetic snapshots that closely captured the experimental data in terms of their high complexity, tight center of mass, and spatially-dependent correlations. Going further, the model was employed to generate a range of new samples that deviated from the data in systematic ways and covered a broad spectrum of conditions where complexity ranged from pathological to healthy states.

Taken together, results suggest that the complexity of population activity provides a marker of neural fluctuations along a continuum of states from healthy to pathologically disinhibited. Furthermore, deep GAN networks offer a promising avenue to study the dynamic control of disinhibited neural activity using brain-computer interfaces with implications for diseases that impact brain networks.

## Results

### Spiral waves

**Fig. 1:**
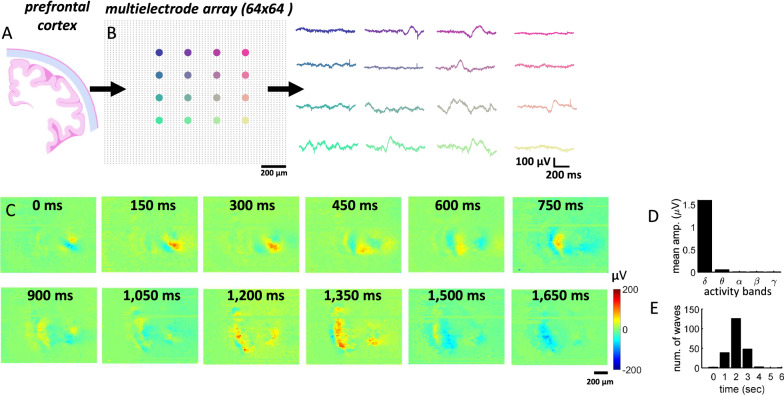
Rotating spiral waves in disinhibited cortical activity. **A** Rodent PFC acute slice recorded with a HD-MEA. **B** Voltage traces across individual channels. Colors correspond to spatial locations of electrodes. **C** Example of spiral wave observed after bath application of PE solution. See movie in Additional file 1. **D** Mean band-filtered voltage across delta (δ, 0–4 Hz), theta (θ, 4–7 Hz), alpha (α, 7–12 Hz), beta (β, 12–30 Hz), and gamma (γ, 30–80 Hz) frequencies. **E** Distribution of spiral wave durations

Activity from coronal prefrontal cortex (PFC) was recorded in acute slices (Fig. 1A) using a HD-MEA after the application of a pro-epileptiform (PE) solution that included 4-Aminopyridine (4-AP) as well as reduced extracellular magnesium (Mg^2+^) and increased extracellular potassium (K^+^). A total of 219 spiral waves were identified across three slices following a set of criteria (see “Methods”). These waves were broadly distributed across electrodes, generating slow fluctuations in activity across recording sites (Fig. 1B). The spatiotemporal evolution of these waves displayed a rotating pattern characteristic of a spiral (Fig. 1C and Additional file 1). While spiral waves were not the only form of activity present in these recordings, they formed a prominent and repeatable pattern over time. Spiral waves were detected at an average rate of 7.3 per minute and their mean voltage amplitude was concentrated in delta frequencies, with lower amplitude found in higher bands (Fig. 1D). The duration of spiral waves was estimated by counting the number of consecutive snapshots (1 ms windows of instantaneous activity) where a wave was identified. The average duration of waves was 2.52 s with standard deviation (SD) of 1.00 s, with both shorter and longer waves present (Fig. 1E). While these values are inherently imprecise due to the manual identification of time windows surrounding spiral waves, they provide an indication that these waves represent slow-evolving events whose timecourse largely exceeds synaptic time constants [28].

By comparison, related work has reported spirals with relatively short durations (< 1 s) [1, 13]. These events, however, were primarily limited to a single cycle, whereas manual inspection of spirals in our data revealed that approximately one third of events had more than a single cycle (one cycle: 63.79%; two cycles: 31.03%; three or more cycles: 3.45% of all spiral waves). The presence of two or more cycles prolonged the duration of spiral event compared with previous accounts and is consistent with in vivo cortical waves [3].

### Center of mass

**Fig. 2:**
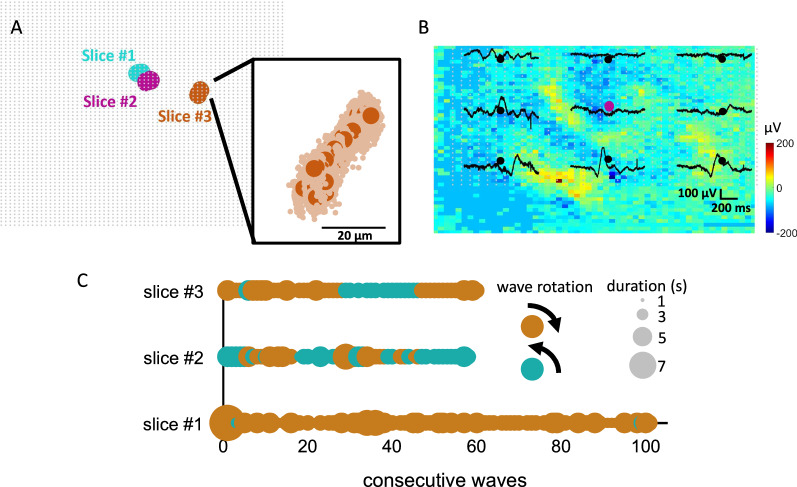
Spatiotemporal attributes of spiral waves. **A** Mean center of mass of individual spiral waves across recordings. Inset shows a zoom of center of mass for spiral waves of a single slice (darker color) and individual time frames (“snapshots”) of each wave (lighter color). **B** Solid black lines are voltage traces at individual electrodes on the array. The center of mass is colored according to slice #2 in panel **A**. **C** Rotational direction and duration of spiral waves across three in vitro cortical slices

Next, the center of mass of each spiral wave was computed by averaging together the central row and column of individual snapshots (“Methods”, Eqs. 1–2). The center of mass was highly consistent across repeated waves of the same slice (Fig. 2A). Variability across waves was primarily delimited to the inter-electrode spacing (20 μm) (Fig. 2A, inset). An example of average voltage activity during a single wave is shown in Fig. 2B. Activity across the network arose in “domains” where groups of neurons were activated over delimited regions of space. Furthermore, voltage activation near the center of mass was lower than surrounding regions [29].

### Direction of rotation

Each spiral wave was assigned a clockwise or counter-clockwise direction of rotation by visual inspection. Overall, 161 waves rotated clockwise and 58 waves counter-clockwise. Because spiral waves may arise by planar waves colliding into each other [1], it is possible that the direction of rotation depends upon the exact arrival times of these simpler waves, which is subject to variability over time. Therefore, we speculated that the angle of rotation may change over the course of a given recording. Consistent with this idea, the direction of rotation alternated across individual waves in two of the slices (Fig. 2C, slices #2 and #3). In these recordings, waves repeated the same rotation several times before switching direction [13]. By comparison, another slice yielded rotational directions that remained mostly consistent over the entire recording (Fig. 2C, slice #1). Thus, cortical networks could exhibit spiral waves with both alternating directions of rotation and waves with more stable patterns characterized by a preferred direction.

### Instantaneous phase

**Fig. 3:**
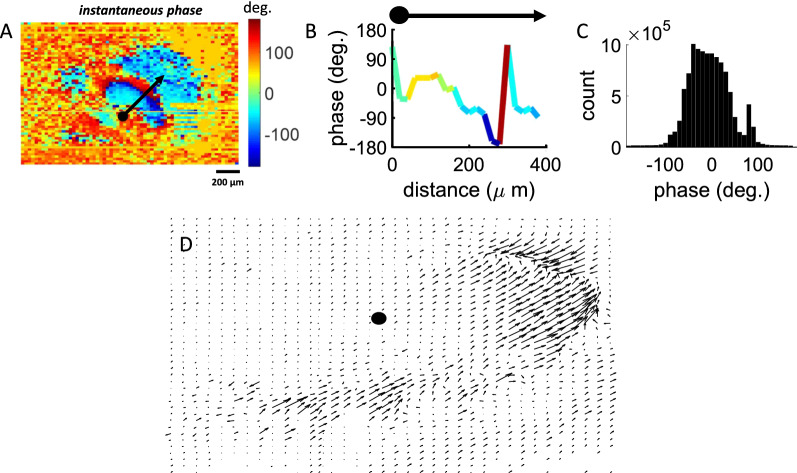
Instantaneous phase of spiral waves. **A** Spatial distribution of instantaneous phases during a rotating wave. Black arrow: direction of vector field used in panel **B**. **B** Instantaneous phase along the vector field in **A**. **C** Global distribution of phases across all spiral waves. **D** Quiver plot showing vector fields of an individual spiral wave calculated between consecutive phase maps separated by 10 ms. Solid black circle: center of mass

Another key feature of spiral waves is the broad distribution of instantaneous phases across individual electrodes [1]. Instantaneous phases were computed by applying a Hilbert transform to delta band-filtered snapshots of activity at a resolution of 1 ms. An example of instantaneous phase obtained at a given time point (Fig. 3A) revealed the presence of a phase gradient radiating from the center of mass of the spiral wave (Fig. 3B). Across all waves, the distribution of instantaneous phases exhibited a broad range of values (Fig. 3C). Thus, snapshots of activity displayed a wide distribution of phases in line with a well-documented signature of spiral waves.

Going further, phase maps were employed to generate vector fields using Matlab’s quiver function. These vector fields indicate the speed and direction of propagating activity across cortical tissue and were employed to validate the presence of spiral waves in segments of neural data [7]. Vector fields are shown by arrows that span a range of orientations representing the flow of spiral waves around a fixed center of mass (Fig. 3D).

### Distance-dependent correlations

**Fig. 4:**
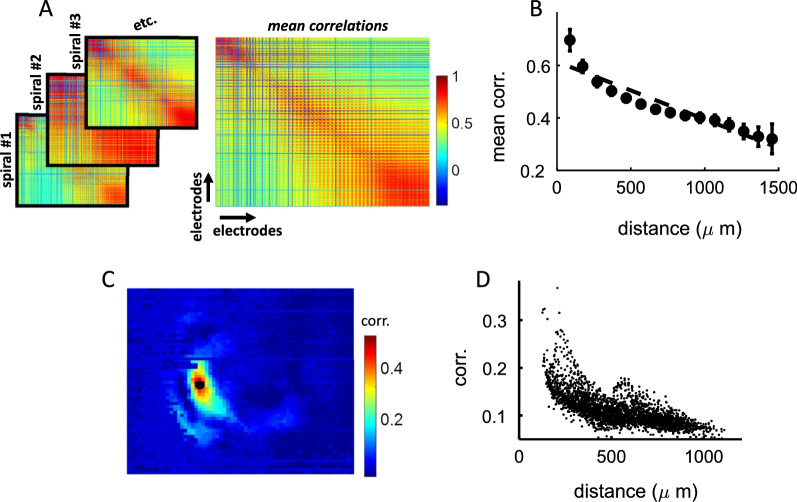
Spatial distribution of correlations during spiral waves. **A** Pairwise correlations were computed for each spiral wave then averaged to create a matrix of mean correlations. **B** The pairwise correlation between electrodes decreased as a function of their spatial distance. Vertical bars: standard error of the mean. Dashed line: best-fitting line of regression. **C** Correlation between the center of mass of a spiral and surrounding electrodes. Filled black circle: center of mass. **D** Correlations relative to the distance from center of mass

Next, network interactions during spiral waves were examined by computing the Pearson correlation between voltages at all pairs of electrodes. Individual correlation matrices were obtained for each spiral of a given network, then averaged to create a mean correlation matrix (Fig. 4A). A widely reported feature of correlations in cortex is their spatial dependence, whereby neighboring cells are on average more strongly correlated than distant pairs [30]. This spatial ordering is also observed in synaptic connectivity where the probability of a monosynaptic contact falls off exponentially with physical distance between neurons [31–33]. Therefore, we reasoned that correlations should decrease with physical distance between pairs of electrodes. Consistent with this prediction, we found a lower mean correlation with increased distance on the array (Pearson correlation test, *R*^2^ = 0.8789, *p* = 2.5193e−07) (Fig. 4B). This analysis was repeated by focusing on the correlation between the center of mass and surrounding points on the array (Fig. 4C). As expected, correlations decreased with increased physical distance from the center of mass (*R*^2^ = 0.3, *p* = 4.5221e−241) (Fig. 4D). Thus, spiral waves displayed distance-dependent interactions consistent with prior findings on functional and structural cortical connectivity.

### Wave complexity

**Fig. 5:**
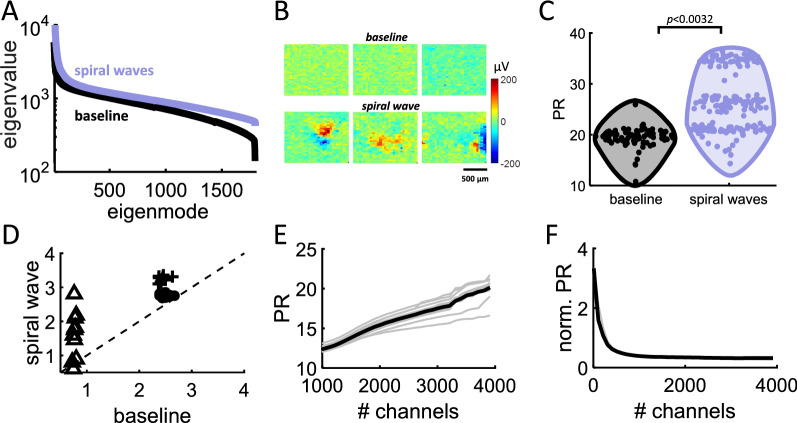
Eigenvalues and complexity of spiral waves. **A** Distribution of ranked eigenvalues for spiral waves in disinhibited slices treated with a PE solution compared to baseline recordings. **B** Examples of snapshots from baseline data vs. spiral wave. **C** Participation ratio of baseline recordings and spiral waves. **D** LBMLE across 10 individual spiral waves and baseline activity of three cortical slices (filled circle, cross, and triangle markers). Dashed line shows unity. **E**, **F** Complexity (PR and normalized PR) versus number of randomly selected multi-electrode channels. Grey lines: individual spirals; solid black line: average over 10 spirals

The complexity of spiral waves was estimated by first applying an eigenspectrum decomposition to population activity, then computing the PR based on the resulting eigenvalues (see “Methods”). Eigenvalues followed a skewed distribution with a broad right tail [25, 34, 35] (Fig. 5A). To evaluate whether complexity was altered in disinhibited cortex, the mean PR of slices was compared before and after application of the PE solution. An equivalent number of snapshots was selected across both conditions (Fig. 5B). The PR across all snapshots yielded a markedly higher value for disinhibited networks compared to baseline (Student’s t-test, *T*_436_ = 2.979, *p* = 0.0032) (Fig. 5C). The average PR value for the baseline was 22.2 (SD: 2.1) compared to 34.31 (SD: 4.24) for spirals. Therefore, spiral waves yielded a higher complexity than baseline, strengthening the view that these waves formed a state of high complexity in cortex [14, 20, 21].

Because the PR is prone to overestimating complexity in neural data [26], the above results were compared to an alternative measure termed the Levina–Bickel maximum likelihood estimation (LBMLE) [36]. This non-linear measure estimates complexity using a geometric approach to calculate the distance between data points. Ten spiral waves and comparable data segments from baseline recordings were selected at random from three cortical slices. For all except one spiral wave, LBMLE complexity was higher with spiral waves than baseline (Fig. 5D). The discrepancy between linear and non-linear measures of complexity is comparable with related work [26]. Hence, both linear (PR) and non-linear (LBMLE) approaches showed that spiral waves yield increased complexity compared to baseline cortical circuits.

Next, we examined how the number of channels $\left( N \right)$ impacted the PR. Random subsets of channels were selected from 10 spiral waves and the PR of those channels was computed. Results show an increase in the PR as the number of selected channels increased (Fig. 5E). This increase could be compensated by scaling the PR by $\sqrt N ,$ resulting in a stable estimate of the PR when at least a few hundred channels were included (Fig. 5F). This effect does not alter our conclusions regarding the increased complexity of spiral waves (Fig. 5C) given that the same number of channels was employed relative to baseline. However, it may be relevant in cases where $N$ varies across conditions.

**Fig. 6:**
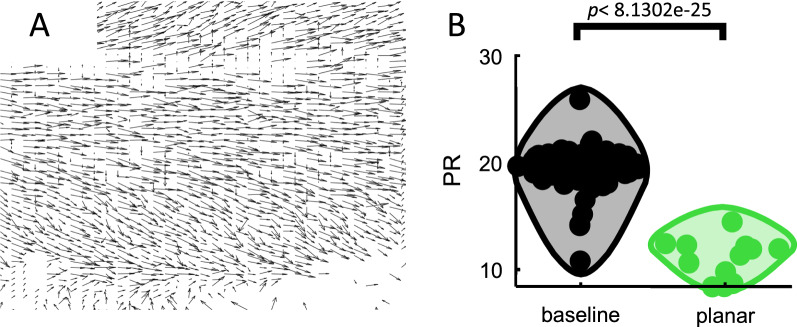
Complexity of planar waves. **A** Quiver plot showing vector fields of an individual planar wave. **B** PR of baseline activity compared to planar waves

Finally, the complexity of baseline activity was compared to planar waves characterized by vector fields that were mainly aligned along a single direction (Fig. 6A). A set of 12 planar waves were manually identified from PE activity. These waves exhibited significantly lower PR than baseline (Student’s t-test, *T*_87_ = 14.4365, *p* = 8.1302e−25) (Fig. 6B). Thus, disinhibited activity was comprised of a mixture of high complexity spiral waves as well as lower complexity planar waves. Other forms of activity, including saddle waves, were likely present but not explicitly detected here.

### Capturing spiral waves in a deep GAN

**Fig. 7:**
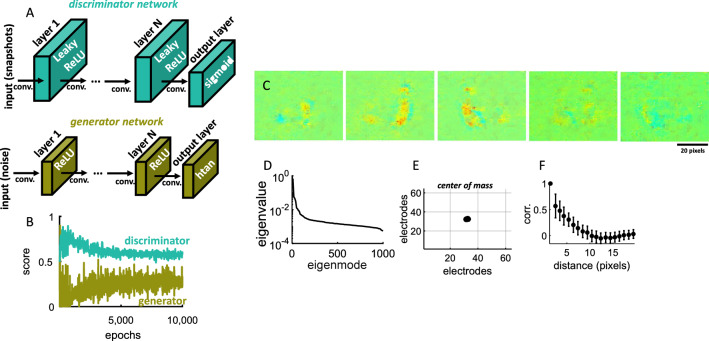
Generative adversarial network trained to capture snapshots of spatial activity. **A** Architecture of the GAN model including both a generator and discriminator network. “conv.”: convolution operator. **B** Performance of the discriminator and generator networks. **C** Snapshots generated by the network after training. **D** Distribution of eigenvalues across 1000 snapshots generated by the network. **E** Center of mass across all snapshots. **F** Pairwise correlations decreased with spatial distance across the GAN snapshots

A deep GAN [27] was trained to produce snapshots that closely matched spiral waves obtained in disinhibited cortical networks (see “Methods”). This model is comprised of a generative network that produces synthetic samples and a discriminator network whose goal is to distinguish between real and synthetic data (Fig. 7A). The GAN was trained for 10,000 epochs, at which point the performance of both the generator and discriminator networks saturated (Fig. 7B).

Once training was completed, noisy input (mean of zero and SD of 25) was injected to the generator network to produce synthetic exemplars of spiral waves (Fig. 7C). A total of 1000 novel snapshots of dimensions 64 × 64 pixels matching the size of the HD-MEA were generated in this fashion. Synthetic snapshots were analyzed similarly to experimental data using their eigenspectrum, center of mass, spatial correlations, and PR.

First, applying an eigenspectrum decomposition to the GAN snapshots yielded a broad distribution of eigenvalues (Fig. 7D) reminiscent of experimental data (Fig. 5A). Second, the center of mass of snapshots was concentrated in a delimited area of space (Fig. 7E) as in experiments (Fig. 2A). Third, spatial correlations were computed across snapshots of individual waves, then averaged together to yield a 4096 × 4096 pixels correlation matrix. As with experimental data, synthetic images had higher correlations for nearby spatial regions (Fig. 7F). This is expected given that the model generated spatially delimited “regions” where activity was highly correlated (Fig. 7C).

**Fig. 8:**
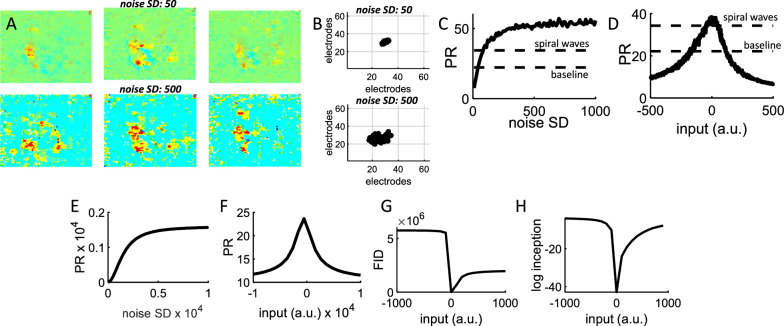
The input provided to generative networks controlled the statistics of snapshots. **A** Examples of snapshots where the SD of the input noise was increased from 50 to 500. **B** Center of mass of 1000 snapshots. **C** The participation ratio increased along with the SD of the input noise. Dashed lines show the participation ratio of baseline and disinhibited cortical activity. 100 images were generated for each value of noise SD. **D** Effect of input strength on the participation ratio of snapshots. Input strength is in arbitrary units (a.u.). **E**, **F** Additive Gaussian noise to a cortical spiral wave altered the PR. **G**, **H** The Frechet Inception Distance (FID) and Inception Score are impacted by the input strength to the GAN. The log of the Inception Score is shown for ease of visualization

Next, a series of analyses examined the PR of snapshots generated by the GAN model. To study a broad range of synthetic images, we varied the SD of the noise injected as input to the generator network. By increasing the noise SD, waves of activity began to break apart into smaller spatial clusters (Fig. 8A) and yielded a more diffuse center of mass (Fig. 8B). Increasing noise SD resulted in higher values of PR, which began to saturate around an SD of 500 (Fig. 8C). PR values obtained from baseline and PE experimental data were included in Fig. 8C as points of comparison, showing that manipulating noise SD yielded a continuum of PRs covering the range of experimental data as well as more extreme cases. Manipulating the mean of the injected noise also yielded a broad range of PR values capturing the scope of experimental data (Fig. 8D).

To compare the results of GAN with experimental data, the effect of noise on PR values was directly assessed by adding Gaussian noise with different means and SD to snapshots of a given cortical spiral wave and computing the resulting PR value. This analysis yielded PR distributions that were qualitatively comparable to those obtained by adding noise to GAN networks. Specifically, noise SD increased the PR until an asymptotic value was reached (Fig. 8E). Further, altering the mean of the Gaussian noise yielded a distribution of PR values that was maximal at zero (Fig. 8F). Hence, GANs provided the ability to not only generate novel samples that were faithful to the statistics of the training data, but also samples that deviated in systematic ways from those statistics. This key feature of GANs could be exploited to study the impact of noise on various measures of neural complexity [26] as well as design brain-computer protocols to study the effects of neurostimulation on epileptiform activity [37].

The performance of the GAN was further assessed using two common performance measures, namely the Inception Score [38] and the Frechet Inception Distance [39]. In both instances, we varied the mean of the noise injected to the GAN and found that better matches to the experimental data were obtained when the noise was near zero (Fig. 8G, H). Hence, the goodness-of-fit of snapshots generated by the GAN was dependent upon the statistics of the noise injected into the network.

**Fig. 9:**
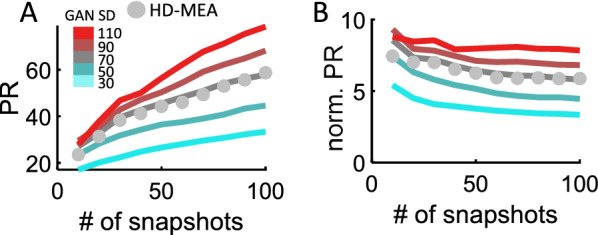
Complexity versus the number of snapshots per spiral wave. **A** GAN approximated HD-MEA data when the SD of its input was 70. **B** Normalizing PR by the square root of the number of snapshots

Finally, we examined how the number of snapshots extracted from each spiral wave affected the PR. For both neural and synthetic data, we extracted a given number of snapshots per spiral and found that increasing the number of snapshots yielded higher values of PR (Fig. 9A). A good fit between the GAN and experimental data was found when the noise injected to the GAN had SD = 70 (Pearson correlation test, *R*^2^ = 0.9795, *p* = 4.9036e−08). Normalizing the PR by the square root of the number of snapshots eliminated most of this effect (Fig. 9B). Thus, while PR is influenced by the number of snapshots, this effect can be largely overcome by normalization and does not alter our conclusions given that the number of snapshots remained constant across conditions.

In sum, the deep GAN model captured key aspects of spiral waves observed in disinhibited cortical networks. Going further, this model was employed to explore a broad range of spatiotemporal activity by manipulating the noise injected as input to the generator network. Below, we discuss the implications of these results for the characterization of pathological network states.

## Discussion

In this work, spiral waves arose in disinhibited cortical networks and exhibited stereotypical characteristics in terms of phase distribution, center of mass, spatial correlations, and neural complexity. Our main finding is that a deep generative neural network produced novel exemplars that captured these characteristics. Further, by adjusting the amplitude and variance of the GAN’s input, the model generated patterns that spanned a broad range of complexity values encompassing both healthy and pathological states of activity.

### Practical applications

There are two main avenues where GANs may be applied to neuropathological activity. First, GANs may inform neurostimulation protocols aimed at the suppression of epilepsy [37]. Results of the GAN network suggest that it may be possible to control the dynamical state and complexity of neural circuits by adjusting the amplitude and variance of injected input. In line with our results, the effect of noise on reducing episodes of neural synchrony has been suggested in theoretical work [40, 41]. In clinical settings, it remains challenging to find regimes of electrical stimulation that are effective at suppressing seizures [42]. This could be addressed by designing generative networks that produce pathological activity, then tuning the input of these networks to optimally suppress this activity. Results of simulations could then be applied to deep brain stimulation and brain–machine interfaces.

A second avenue of application for GAN models is the generation of large datasets of plausible exemplars from a known distribution. This is an important application given that certain brain events such as seizures occur infrequently but are key to understanding the underlying neural pathology. The current work is a prime example of such application, where a GAN was employed to generate a dataset of spiral waves that are relatively rare in cortical recordings. This dataset can then be employed to examine the robustness of key properties of neural activity and train decision-based systems that serve as diagnostic aid [43–45].

### Related approaches

Our work can be compared to approaches that fall into two categories, namely generative models and biologically-inspired networks. Increasingly sophisticated generative models have emerged in recent years, with the capability to produce realistic images [46–49] and videos [50–54]. Few studies, however, have applied GANs to brain data [55–58], and none thus far have looked at epileptiform brain activity.

Biologically-inspired models have been successful at capturing UP-DOWN states of rhythmic activity [59–61] as well as spiral waves [1, 37, 62–64]. A key advantage of these models is that they suggest candidate neural mechanisms to produce spiral waves. Notably, waves are proposed to emerge via three main scenarios: (i) an initially localized oscillation that propagates through lateral interactions; (ii) a shared input that drives nearby cortical sites with different transmission delays; and (iii) several sites that oscillate at similar frequencies and form coherent patterns [62]. Biologically-inspired models, however, are not designed to function as generative models that capture the statistics of a given dataset. A hybrid approach will hopefully emerge where biologically-inspired GANs can serve as data generators while embodying biological principles. Ideally, this approach would allow GANs to behave as a dynamical system that captures the mechanisms involved in generating seizure activity.

While our work employed PR and LBMLE as measures of complexity, various linear and non-linear alternatives have been proposed [26]. While non-linear approaches may provide a more accurate estimation of complexity, it is unclear what method best applies to disinhibited neural data compared across experimental conditions. A complete theoretical analysis of PR and related measures will be needed to shed light on the relation between noise, disinhibited activity, and neuronal complexity.

### Measures of neural complexity

Several measures of neural complexity have been proposed [26]. Linear methods such as the PR are widely used and straightforward to interpret due to their simplicity. However, linear methods tend to overestimate the dimensionality of neural data. Hence, we compared the PR to a non-linear LBMLE method (Fig. 5D). With both approaches, results consistently showed that spiral waves led to an increase in complexity compared to baseline activity. Another factor to consider is that measures of complexity such as the PR scale with the number of channels $\left( N \right)$ analyzed (Fig. 5E) and the resolution (i.e., number of snapshots) of the data (Fig. 9A). This does not affect our main conclusions given that the number of neurons and snapshots was constant across spiral waves and baseline conditions. However, for applications where the number of channels and resolution may vary, it would be useful to scale the PR by these values. This will yield more stable estimates of complexity (Figs. 5F, 9B).

### Alternatives to GANs

While GANs were successful at capturing several aspects of spiral waves and hold the state-of-the-art for image generation, it is worth considering the advantages and drawbacks of potential alternatives, including diffusion models [65], variational autoencoders [66], and U-nets [67]. Diffusion models are a class of likelihood-based models that have recently been shown to produce high-quality images and avoid the “collapse” problem associated with GANs that produce images within a limited range of the training space. These models, however, tend to be slower and require more user intervention, in the form of classifier guidance. Variational autoencoders process input data by reducing it to a latent space of lower dimensionality prior to reconstruction. Results are generally inferior in quality than GANs. Finally, U-net is a generative model that uses a segmentation network as the discriminator, where the goal is to partition an image into several basic constituents. A restriction of this approach, however, is that the input and output dimensions of the network must be the same. How these different generative models compare when trained on neural data is an interesting question for future work.

### Comparison to in vivo spiral waves

Despite the in vitro nature of the data analysed herein, our results share several characteristics of spiral waves found in vivo during sleep-like states [3], epileptic activity [13], and anaesthesia [6]. These characteristics include a broad phase distribution, a low amplitude near the center of mass, and the co-occurrence of spiral waves with other forms of activity including planar waves. The advantage of an in vitro approach using an HD-MEA is the ability to monitor spiral waves using a large number of channels simultaneously. The resulting data allowed us to elucidate several aspects of spiral waves that had not previously been explored, including spatial correlations and complexity. These results will benefit from in vivo support in future studies.

### Limitations and future work

While our results suggest increased complexity in disinhibited cortical networks, it is unclear whether these results would generalize to surrounding brain regions. In hippocampus, for instance, chaotic dynamics were mainly confined to the dentate gyrus and subiculum, while lower levels of chaotic activity were found in areas CA1–CA4 [20]. It would be worthwhile to explore seizure-like activity across brain regions and capture their differences using generative networks.

Furthermore, disinhibited networks produce various forms of waves that have not been explored here, including saddle patterns formed by the interaction between multiple waves [2–8]. Future work should be aimed at capturing the diversity of waves produced during healthy and disinhibited cortical states.

Caution should be warranted when attempting to draw general conclusions about neural complexity based strictly on spiral waves without also considering other forms of neural events as well as inter-wave activity. Spiral waves are interleaved with other neuronal patterns, including periods of both synchronized and desynchronized activity [4]. It is possible that analyzing spiral waves in isolation may suggest increased neural complexity, while a broader range of activity may reveal otherwise. Here, we focused on spiral waves as they constitute an intricate form of neural activity that has thus far eluded a complete characterization. More broadly, neural complexity remains poorly understood as it covaries with many factors including cognitive attention [14], task demands [68, 69], arousal state [70], and neural pathologies [22].

Finally, the prospects of using artificial neural networks to monitor and dynamically control epileptic events in real time will require the implementation of GANs that can handle continuous input streams and produce time-evolving synthetic data. This field of research is currently under development and requires a combination of GANs with recurrent neural networks [71, 72].

## Conclusions

During states of disinhibited activity, cortical circuits generate propagating waves whose spatial and temporal evolution follows reliable patterns [1]. A deep generative neural network trained on cortical spiral waves captured key aspects of these patterns. Once trained, the model was employed to show that neural complexity varies along a continuum—from lower values in healthy states to higher values in disinhibited states. The complexity of the simulated data was achieved solely by controlling the amplitude and variance of the input fed to the model, suggesting a framework that can be employed to examine the stimulus-driven suppression of aberrant network activity. This work opens the door to novel approaches that derive synthetic exemplars from neuroscience data to study rare forms of activity and probe their causal origins.

## Methods

### Electrophysiological data collection

#### Animals

All data were collected using three Sprague Dawley rats of both sexes (2 males and 1 female), aged 14 to 21 days, purchased from Charles River. Animals were housed in standard housing conditions with cage enrichment and ad libitum access to water and standard chow. All experiments were conducted in accordance with the Canadian Council on Animal Care guidelines and all procedures were approved by the University of Ottawa Animal Care and Veterinary Services.

#### Acute slice preparation

Animals were deeply anaesthetized using isofluorane (Baxter Corporation) and subsequently euthanized via decapitation. Brains of the animals were quickly extracted and submerged into a frozen choline dissection buffer. The buffer consisted of the following: 119.0 mM choline chloride, 2.5 mM KCl, 4.3 mM MgSO_4_, 1.0 mM CaCl_2_, 1.0 mM NaH_2_PO_4_, 1.3 mM sodium ascorbate, 11.0 mM glucose, 26.2 mM NaHCO_3_, and was perfused using carbogen (95% O_2_/5% CO_2_). Acute cortical slices containing the PFC were produced using a Leica VT1000S vibratome. The brain was sliced coronally at a thickness of 300 µm. Once the slices were collected, they were placed in a recovery chamber filled with a standard artificial cerebrospinal fluid (ACSF) consisting of 119.0 mM NaCl, 2.5 mM KCl, 1.3 mM MgSO_4_, 2.5 mM CaCl_2_, 1.0 mM NaH_2_PO_4_, 11.0 mM glucose, and 26.2 mM NaHCO_3_. The ACSF was continuously perfused using carbogen (95% O_2_/5% CO_2_) and maintained at a temperature of 37 °C. Following slicing, the chamber was left to recover for 1 h prior to experiments where it equilibrated to room temperature.

### Multi-electrode arrays

#### Generation of epileptiform activity

Baseline data was recorded using standard ACSF prior to application of the PE solution. Slices were included in the study if they displayed neural activity during baseline recordings, defined as threshold-crossing events in voltage traces on the acquisition software. Following baseline recordings, epileptiform activity was generated by applying a pro-epileptiform ACSF (PE-ACSF) containing the following: 120 mM NaCl, 8.5 mM KCl, 1.25 mM NaH_2_PO_4_, 0.25 mM MgSO_4_, 2 mM CaCl_2_, 24 mM NaHCO_3_, 10 mM dextrose, and 0.05 mM 4-AP [73]. The PE-ACSF included a potassium channel blocker (4-AP) as well as reduced extracellular magnesium (Mg^2+^) and increased extracellular potassium (K^+^), all of which have been reported to induce epileptiform activity [73–79] and increase synchronization [80, 81]. The PE-ACSF solution was applied for 20 min prior to beginning the recordings and epileptiform activity was recorded for 10 min.

#### Multi-electrode recordings

Extracellular potentials were collected using an active pixel sensor HD-MEA. This array uses a complementary metal-oxide semiconductor monolithic chip in which the pixels were modified to detect changes in electric voltages from electrogenic tissue. The circuit is designed to provide simultaneous recordings from 4096 electrodes with a sampling rate of 7.7 kHz per channel. The chips are comprised of 64 × 64 electrodes arranged as a pixel element array whereby each pixel measures 21 μm × 21 μm with an electrode pitch of 42 μm. The active area of the array is 7.22 mm^2^ and has a pixel density of 567 pixels/mm^2^ [22, 82]. Data were acquired using BrainWave software (3Brain Gmbh, Switzerland) and imported to Matlab (MathWorks, Natick) for offline analysis.

### Identification of spiral waves

Voltages at individual channels were processed by first applying a second order bandpass Butterworth filter in the delta range (1–4 Hz) to the raw voltages in the forward and reverse directions using the filtfilt function in Matlab [56, 83, 84]. Artefacts were removed by setting time-points with absolute values greater than 200 μV to the mean of the signal. Data segments containing spiral waves were extracted based on visual inspection and later verified by the following criteria [29]: (i) a broad distribution of instantaneous phases around the center of mass (Fig. 3A–C); (ii) rotating vector fields (Fig. 3D); (iii) a decrease in voltage near the center of mass (Fig. 2B); and (iv) spatially-dependent correlations between pairs of channels (Fig. 4).

#### Center of mass

The center of mass of a given spiral wave was obtained as follows [85, 86]. Assuming a 64 × 64 array of elements $a_{ij}$ reflecting the band-filtered voltage at a particular time and spatial location (row $i$ and column $j$ up to $N$ electrodes), the center row $\left( r \right)$ and column $\left( c \right)$ are given by

1 $$ r = \frac{{\sum\nolimits_{i,j}^{N} {i \cdot a_{ij} } }}{{\sum\nolimits_{i,j}^{N} {a_{ij} } }}, $$

and

2 $$ c = \frac{{\sum\nolimits_{i,j}^{N} {j \cdot a_{ij} } }}{{\sum\nolimits_{i,j}^{N} {a_{ij} } }}. $$

The above expressions were computed for each 1 ms time frame (“snapshot”) of a given spiral wave, then averaged to provide the mean center of mass of each wave.

#### Complexity

The complexity of a given spiral wave was estimated by applying an eigenspectrum decomposition [87, 88] to 6 evenly-spaced snapshots of each spiral wave, yielding ranked eigenvalues $\lambda_{1} , \ldots ,\lambda_{N}$ where $N$ is the total number of channels. Then, complexity was calculated using the PR [23–25],

3 $$ {\text{PR}} = \frac{{\left( {\sum\nolimits_{i}^{N} {\lambda_{i} } } \right)^{2} }}{{\sum\nolimits_{i}^{N} {\lambda_{i}^{2} } }}, $$

corresponding to the square of the eigenspectrum’s first moment normalized by its second moment. If patterns of neural activity are limited to a few dimensions, only a few eigenvalues will be positive, and the PR will be low. However, more complex, high-dimensional neural activity will be reflected by a broad distribution of eigenvalues and a high PR value.

### Generative adversarial network

In the GAN framework, two artificial neural networks compete against each other [27]. The “generative model” (*G*) attempts to produce synthetic samples that closely match the original data, while its counterpart, the “discriminative model” (*D*), learns to discriminate these synthetic samples from genuine ones. The competition between these two networks drives the GAN to produce synthetic samples that are indistinguishable from the original data. Once successfully trained, novel samples can be obtained from the generative model by feeding random noise to its input layer.

Here, a GAN was trained to produce synthetic samples of spiral waves. Once cortical spiral waves were identified and verified based on the above criteria, a sample of 6 snapshots were collected per spiral, corresponding to evenly spaced time points between the approximate time of initiation and termination of the wave. The complete dataset consisted of 1314 images obtained from 219 spiral waves. Each input to the GAN consisted of all 6 snapshots from an individual wave tiled to form a pattern of size 64 pixels × 64 pixels × 6 snapshots.

Formally, assume some real data $\left\{ {x^{\left( i \right)} } \right\}_{i = 1}^{m} \sim {\mathbb{P}}_{r} ,$ where ${\mathbb{P}}_{r}$ is the data distribution. The goal was to generate some novel data $\tilde{\user2{x}}$ whose distribution ${\mathbb{P}}_{g}$ is a close approximation of ${\mathbb{P}}_{r}$. This was achieved by feeding noise to the generator network, $\tilde{\user2{x}} = G_{\theta } \left( z \right),$ given noisy priors $\left\{ {z^{\left( i \right)} } \right\}_{i = 1}^{m} \sim p\left( z \right)$. The input $z$ to the generator was sampled from a Gaussian distribution.

The generative and discriminative networks were trained according to a minimax objective function,

4 $$ \mathop {\min }\limits_{G} \mathop {\max }\limits_{D} V\left( {D,G} \right) = \,{\mathbb{E}}_{{{\varvec{x}}\sim p_{data} \left( {\varvec{x}} \right)}} \left[ {logD\left( {\varvec{x}} \right)} \right] + {\mathbb{E}}_{{{\varvec{z}}\sim p_{{z\left( {\varvec{z}} \right)}} }} \left[ {\log \left( {1 - D\left( {G\left( {\varvec{z}} \right)} \right)} \right)} \right], $$

where $V\left( {D,G} \right)$ is a min–max value function and ${\varvec{x}}$ is the original data. This objective function was optimized using the Adam optimizer [89] with a discriminator network learning rate of $\alpha$ = 0.0002. The generator network learning rate was $\alpha$ = 0.001. The total number of training iterations was set to 10,000. The generator network was composed of six hidden layers with rectified linear units (ReLU) and a hypertan (htan) output layer. The discriminator network had eight hidden layers with leaky ReLU units and a htan output layer. A convolution step preceded each hidden layer. The full model was trained using the Matlab Deep Learning library with default parameters unless otherwise stated. Output images were 64 × 64 pixels in size, matching the dimensions of the input snapshots obtained from the HD-MEA.

The performance of the generator network (Fig. 7B) was computed by the score

5 $$ S_{G} = mean\left( {\hat{Y}_{generated} } \right), $$

where $\hat{Y}_{generated}$ contains the probabilities for the generated images. For the discriminator network, the score was

6 $$ S_{D} = 0.5 mean\left( {\hat{Y}_{real} } \right) + 0.5 mean\left( {1 - \hat{Y}_{generated} } \right), $$

where $\hat{Y}_{real}$ contains the discriminator output probabilities for real images. The ideal scenario is where both scores are close to 0.5. However, this is not a requirement to obtain a successful GAN; in fact, several measures were employed to compare the generated images with experimental data, including eigenspectrum distribution (Fig. 7D), center of mass (Fig. 7E), spatial correlations (Fig. 7F), complexity (Fig. 8C–F), Frechet Inception Distance (Fig. 8G), and Inception Score (Fig. 8H).

## Supplementary Information

**Additional file 1.** Movie showing an example of a single spiral wave recorded with a HD-MEA. Color map shows voltages ranging between [− 200, 200] μV. Each frame of the movie was a snapshot of 100 ms.

## Data Availability

The datasets used and/or analyzed during the current study are available from the corresponding author upon reasonable request.

## Abbreviations

PFC: Prefrontal cortex
PE: Pro-epileptiform
ACSF: Artificial cerebrospinal fluid
PE-ACSF: Pro-epileptiform artificial cerebrospinal fluid
HD-MEA: High-density multi-electrode array
GAN: Generative adversarial network
ReLU: Rectified linear units
htan: Hypertan
SD: Standard deviation
PR: Participation ratio
LBMLE: Levina–Bickel maximum likelihood estimation
